# Do Postures of Distal Effectors Affect the Control of Actions of Other Distal Effectors? Evidence for a System of Interactions between Hand and Mouth

**DOI:** 10.1371/journal.pone.0019793

**Published:** 2011-05-23

**Authors:** Maurizio Gentilucci, Giovanna Cristina Campione

**Affiliations:** 1 Department of Neuroscience, University of Parma, Parma, Italy; 2 Brain Center for Social and Motor Cognition, Italian Institute of Technology, Parma, Italy; French National Centre for Scientific Research, France

## Abstract

The present study aimed at determining whether, in healthy humans, postures assumed by distal effectors affect the control of the successive grasp executed with other distal effectors. In experiments 1 and 2, participants reached different objects with their head and grasped them with their mouth, after assuming different hand postures. The postures could be implicitly associated with interactions with large or small objects. The kinematics of lip shaping during grasp varied congruently with the hand posture, i.e. it was larger or smaller when it could be associated with the grasping of large or small objects, respectively. In experiments 3 and 4, participants reached and grasped different objects with their hand, after assuming the postures of mouth aperture or closure (experiment 3) and the postures of toe extension or flexion (experiment 4). The mouth postures affected the kinematics of finger shaping during grasp, that is larger finger shaping corresponded with opened mouth and smaller finger shaping with closed mouth. In contrast, the foot postures did not influence the hand grasp kinematics. Finally, in experiment 5 participants reached-grasped different objects with their hand while pronouncing opened and closed vowels, as verified by the analysis of their vocal spectra. Open and closed vowels induced larger and smaller finger shaping, respectively. In all experiments postures of the distal effectors induced no effect, or only unspecific effects on the kinematics of the reach proximal/axial component. The data from the present study support the hypothesis that there exists a system involved in establishing interactions between movements and postures of hand and mouth. This system might have been used to transfer a repertoire of hand gestures to mouth articulation postures during language evolution and, in modern humans, it may have evolved a system controlling the interactions existing between speech and gestures.

## Introduction

Arm actions, and in particular the grasp of objects, are among the most refined activities of primates. They require highly specialized nervous structures for their planning and control. In monkeys the circuit formed by the anterior part of the intraparietal sulcus (AIP) [1] and F5 premotor area [2] is involved in the control of grasp movements [3]. Sakata and colleagues [1] proposed that AIP extracts from the objects the properties affording appropriate interactions with them. These informations are then relayed to F5 for selection of the type of grasp and the pattern of grasp movements (the affordance) [4]–[5].

The F5 premotor circuit also plays a role in coding more cognitive aspects of grasp [6]. In particular, Rizzolatti and colleagues [3] recorded F5 neurons involved in commanding grasp motor acts with either the hand or the mouth. These neurons seem to code the goal of the grasp, i.e. taking possession of an object. According to the idea that spoken language derives, at least partially, from a primitive communication system based on arm gestures [7]–[16], Gentilucci and colleagues [17]–[19] suggested that during evolution, a system derived from F5 premotor area, where neurons commanding grasps with both hand and mouth were recorded, could have been used to transfer the repertoire of hand gestures to mouth articulation postures. In modern humans, a system of double motor commands to hand and mouth may also be involved in controlling the interactions existing between speech and gestures [20]–[21] and it may be located in Broca's area [22]. This view seems to be congruent with the Rizzolatti and Arbib's hypothesis [15]: these authors, on cytoarchitectonic and functional grounds, proposed that Broca's area derives phylogenetically from F5 premotor area. Broca's area, and in particular area BA44, is anatomically adjacent to premotor area and it is thought to be involved in encoding phonetic representations in terms of mouth articulation gestures [23]–[25].

In humans, evidence of the activity of a system of double hand-mouth motor commands come from a behavioral study by Gentilucci and colleagues [26]. In particular, in one experiment of their kinematic study [26] participants were required to reach and grasp small and large objects with their hand while simultaneously opening their mouth by a fixed amount. Conversely, in another experiment participants were required to reach small and large objects with their head and to grasp them with their mouth while simultaneously opening their thumb and index finger by a fixed amount. The authors found that mouth and finger opening were affected by finger and mouth shaping during grasp. Specifically, they were larger when grasping large as compared to small objects. However, the results of these two experiments leave the following issues unsolved. First, Gentilucci and colleagues [26] found that the grasp executed with one effector (hand or mouth) affected the posture assumed from the other effector (mouth or hand). However, the reverse was not verified. Specifically, the authors did not verify whether previously assumed postures of one effector (mouth or hand) which may be implicitly related to different interactions with objects, affect the control of the successive grasp executed with the other distal effector (hand or mouth). An affirmative response to this question suggests that a posture of a distal effector is sufficient to affect the control of the movement of another distal effector, such as the movement of a distal effector affected the posture of another distal effector [26]. This, in turn, might support the hypothesis that the system involved in the interactions between distal postures and grasp actions might be the precursor of a system involved in the interactions between gesture and speech [20]–[21]. Indeed, speech and gestures are produced by both postures and movements of the corresponding effectors. In addition, the present study may exclude that interactions between two distal effectors are only due to synchronisms between their movements. In fact, in the study by Gentilucci and colleagues [26], the grasping with an effector was simultaneous to the movement of the other effector when assuming a posture (i.e. the opening of the fingers or the mouth). In contrast, in the present study, the postures of an effector were assumed before the initiation of the grasp with the other effector. Second, Gentilucci and colleagues [26] did not verify whether the reciprocal interactions between postures and actions were specific for hand and mouth or they could be extended to the other distal effector, namely the foot.

We addressed these issues in experiments 1, 2, 3 and 4. In experiments 1 and 2, we searched for effects of hand postures on grasps with the mouth. In experiment 1 the hand posture took into account both finger flexion/extension, and thumb opposition to the other fingers. These postures pantomimed the interaction with large (power grip) and small objects (precision grip). In contrast, in experiment 2, they took into account finger extension/flexion only. These latter postures were chosen to make the hand postures comparable to those taken by the mouth (jaw lowering/lifting) and the foot (extension/flexion of toes) in experiments 3 and 4, respectively. Indeed, in experiments 3 and 4, we searched for effects of mouth and foot postures on grasps with the hand, respectively. We used open and closed mouth as mouth postures in experiment 3 and toe extension and flexion as foot postures in experiment 4.

In one experiment of the study by Gentilucci and colleagues [26], participants reached and grasped small and large objects with their hand while pronouncing a syllable. The grasp affected syllable pronunciation, whereas the reverse was not observed. This lack of an effect could be due to the fact that pronunciation of the syllable was successive to the grasp beginning, and duration of syllable pronunciation was briefer than duration of grasp. Consequently, the syllable could have poor access to the grasp at the level of planning and/or control of movement execution. For these reasons, in experiment 5 we reexamined the possibility that speech affects the grasp by requiring participants to vocalize and then to grasp objects of different size while continuing to vocalize.

## Experiment 1

Participants reached with their head and grasped with their mouth either a large or a small piece of food after assuming a hand posture pantomiming either a power grip (i.e. a type of interaction with a large object) or a precision grip (i.e. a type of interaction with a small object). We expected an effect of hand posture on mouth shaping, that is a larger mouth shaping after assuming a hand power grip, and a smaller mouth shaping after assuming a hand precision grip.

### Methods

#### Participants

Ten right-handed [27], naïve volunteers (7 females and 3 males, age 23–30 yrs.) participated in the experiment. The Ethics Committee of the Medical Faculty at the University of Parma approved the study, which was carried out according to the declaration of Helsinki. We obtained written informed consent from all participants in the present study.

#### Apparatus, Stimuli, and Procedure

**Figure 1:**
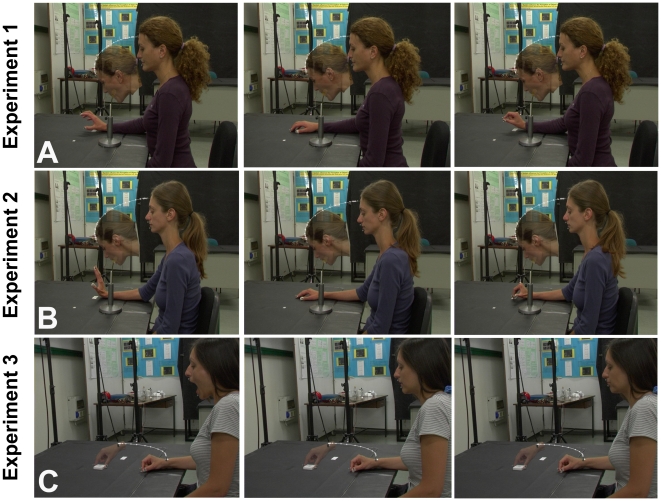
Experimental set-up, stimuli, procedure and examples of trajectories in experiments 1–3. A. Experimental set-up, stimuli, procedure and examples of the head reach and mouth grasp performed by the participants in experiment 1. White lines represent examples of head trajectories. In the left, central, and right panel the conditions of power grip posture, relaxed hand posture and precision grip posture are presented, respectively. B. Experimental set-up, stimuli, procedure and examples of the head reach and mouth grasp performed by the participants in experiment 2. White lines represent examples of head trajectories. In the left, central, and right panel the conditions of extended finger posture, relaxed finger posture and flexed finger posture are presented, respectively. C. Experimental set-up, stimuli, procedure and examples of the hand reach-grasp performed by the participants in experiment 3. White lines represent examples of hand trajectories. In the left, central, and right panel the conditions of open, relaxed, and closed mouth posture are presented, respectively. The participants shown in the panels have seen this manuscript and figures and has provided written consent for publication.

The participants sat in front of a table on which they placed their right hand. Stimuli were two parallelepiped-shaped candies (small target: 1.0×1.0×2.0 cm; large target: 1.5×1.5×3.0 cm). One candy was placed on a support located on the table plane. The candy was approximately 24 cm distant from the mouth of the participant when standing in starting position (Fig. 1A). The participants, whose mouth was closed at trial beginning, were required to reach the candy with their head and to grasp it with their mouth (Fig. 1A); they were required to move with a natural velocity as during spontaneous movements. The actions were executed in the three following experimental conditions during which the right hand posture randomly changed: power grip posture, relaxed hand (i.e. control) posture and precision grip posture (Fig. 1A). In the power grip posture the fingers were extended and the thumb was in opposition to the other fingers. In the precision grip posture the finger were flexed and the thumb was in opposition to the index finger. Before every trial, the participants, whose eyes were closed, were required to take one of the three hand postures. When they were confident to have taken the correct posture, they were required to open their eyes and to start the reach to grasp action, maintaining that hand posture during the entire action. Consequently, the hand posture was assumed without any visual control of the effector, as it occurred in the other experiments of the present study. However, during the head movements, the participants could see their hand with peripheral vision. The three experimental conditions were randomly presented in the same session; for each condition 16 trials were run (in half of the trials the large candy was presented, and in the remaining trials the small one with a random order). In total, 48 trials were run.

#### Data Recording

Movements of the participants' mouth and postures of their right hand were recorded using the 3D-optoelectronic SMART system (BTS Bioengineering, Milano, Italy). This system consists of six video cameras detecting infrared reflecting markers (spheres of 5-mm diameter) at a sampling rate of 120 Hz. Spatial resolution of the system is 0.3 mm. Recorded data were filtered using a linear smoothing low pass filter, i.e. a triangular filter where each value was the weighted mean computed over 5 samples (window duration: 33.3 ms).

We used three markers attached to the upper lip, lower lip and to the forehead of the participants. Another two markers were attached, one to the thumb, and one to the index finger of the participant's right hand. The markers placed on the upper and lower lip were used to study the kinematics of mouth grasp. Starting from a posture of lip closure, mouth grasp time course is constituted by a lip opening phase until a maximum (maximal lip aperture) followed by a phase of lip closing on the object [26]. We analyzed peak velocity of lip opening, and maximal lip aperture. The kinematics of the marker placed on the forehead was used to study the head reach. We analyzed head reach peak velocity. The method for calculating the beginning and end of reach and grasp is described elsewhere [4]. We used the markers placed on the thumb and the index finger to measure the mean finger aperture during the head reach, i.e. from the reach beginning to the reach end. Due to technical problems during acquisition, data for one participant were discarded.

#### Data analysis

Separate ANOVAs were carried out on the mean values of the mouth reaching-grasping parameters and finger aperture. The within-subjects factors were target size (large versus small) and hand posture (power grip versus relaxed hand versus precision grip). In all analyses post-hoc comparisons were performed using the Newman-Keuls procedure.The significance level was fixed at p = 0.05. When a factor or the interaction between factors were significant, we also calculated the effect size [η^2^_p(artial)_].

### Results and Discussion

**Figure 2:**
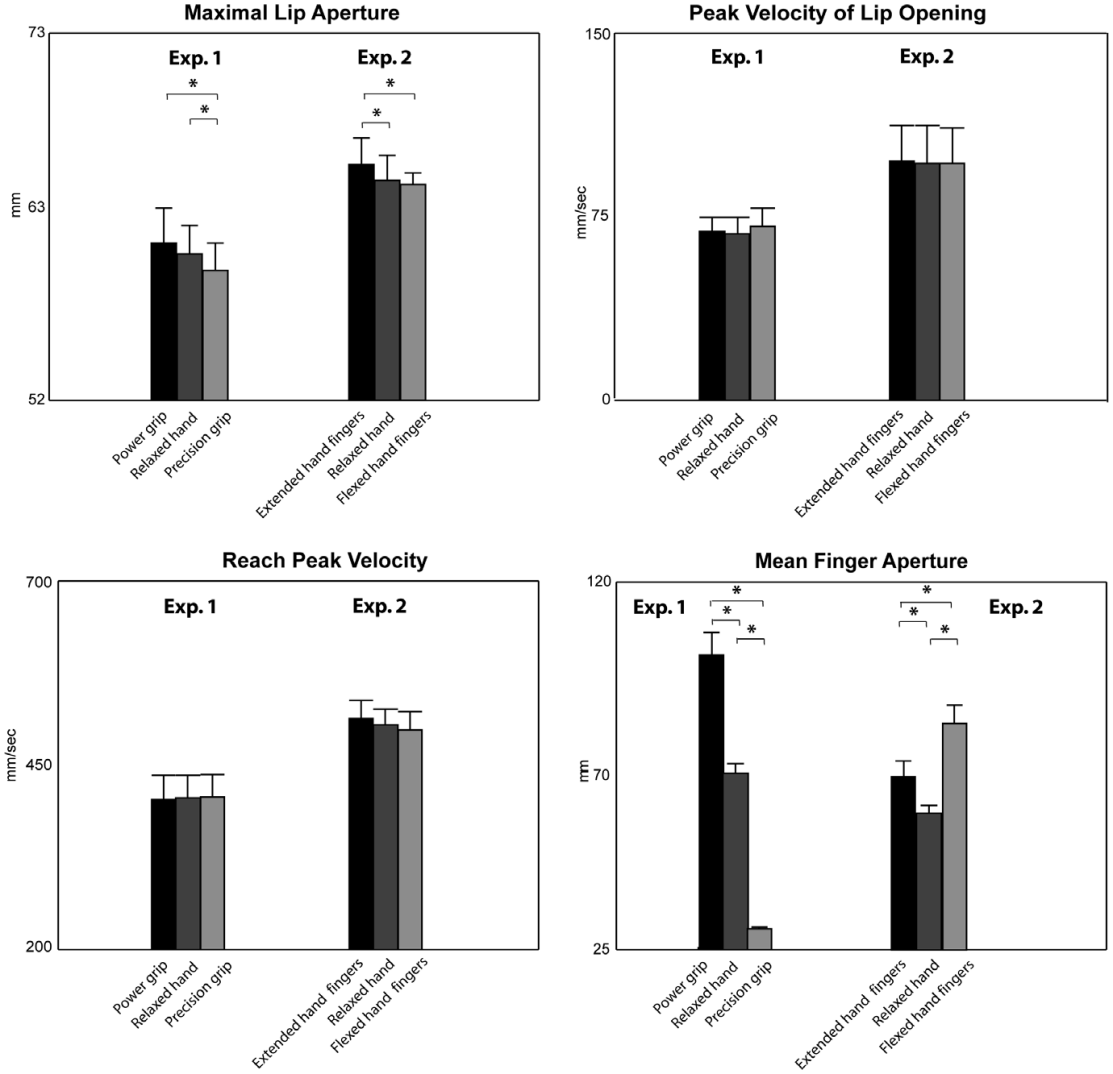
Mean values of kinematic parameters of mouth grasp, head reach and mean finger aperture in the three experimental conditions of hand posture, of experiments 1 and 2. Bars are SE. Asterisks indicate significance in the ANOVAs.

The main results are the following. Maximal lip aperture was affected by hand posture (F(1, 9) = 6.2, p<0.001, η^2^_p_ = 0.41). This parameter was greater in the conditions of power grip and relaxed hand as compared to the condition of precision grip (Fig. 2, post-hoc comparison). Mean finger aperture significantly increased moving from precision grip to power grip posture (F(1,8) = 78.4, p<0.0001, η^2^_p_ = 0.90, Fig. 2, post-hoc comparison). The other results are reported in Table S1 (Results of the ANOVAs on kinematic parameters of reaching and grasping executed with the mouth while the hand is in a power grip posture, is relaxed, and is in a precision grip posture).

The results of experiment 1 support the hypothesis that hand postures pantomiming a power and a precision grip influenced mouth shaping during a successive grasping with the mouth. Specifically, maximal lip aperture was larger after assuming a power grip posture, whereas it was smaller after assuming a precision grip posture.

The hand postures taken in the present experiment took into account both finger flexion/extension, and thumb opposition to the other fingers. In experiments 3 and 4 we studied the effects of mouth closure/aperture (jaw lifting/lowering) and toe extension/flexion. In order to make the posture of the hand comparable with those of the mouth and foot, in experiment 2, we required participants to extend/flex the hand fingers before grasping the food with their mouth.

## Experiment 2

### Methods

#### Participants

A new sample of ten right-handed [27], naïve volunteers (9 females and 1 males, age 26–30 yrs.) participated in the experiment.

#### Apparatus, Stimuli, and Procedure

Apparatus, stimuli and procedures were the same as in experiment 1. However, the three hand postures assumed by the participants were different from those assumed in experiment 1. They were the following: extended hand fingers 2–5, relaxed hand fingers, and flexed hand fingers 2–5. In the two conditions of flexed/extended fingers, the thumb was in a posture of non-opposition to the other fingers or the hand palm (Fig. 1B).

#### Data Recording and Analysis

Movement recording and analysis were the same as in experiment 1. In the ANOVAs the within-subjects factors were target size (large versus small) and hand posture (extended hand fingers versus relaxed hand versus flexed hand fingers).

### Results and Discussion

Maximal lip aperture was greater in the conditions of extended hand fingers as compared to the conditions of relaxed hand fingers and flexed hand fingers (F(1, 9) = 3.9, p<0.05, η^2^_p_ = 0.30, Fig. 2, post-hoc comparison). Mean finger aperture was significantly different in the three conditions of hand posture (F(1, 8) = 12.6, p<0.0001, η^2^_p_ = 0.58, Fig. 2, post-hoc comparison). Note in Figure 2 that the variation in lip aperture could be not associated with spatial relations between thumb and index finger or hand palm (thumb opposition). The other results are reported in Table S2 (Results of the ANOVAs on kinematic parameters of reaching and grasping executed with the mouth while the hand fingers are extended, relaxed and flexed).

The results showed that mouth shaping increased when the hand fingers were extended, whereas it decreased when the hand fingers were flexed. In experiment 2 the relaxed hand finger posture had an effect on grasp not significantly different from that of the flexed hand finger posture, whereas in experiment 1 it had an effect not significantly different from that of the extended hand finger posture. These results may be explained as follows: in experiment 1 the posture of relaxed hand could be more easily associated with the power grip posture than the precision grip posture (Fig. 1A), whereas in experiment 2 it could be more easily associated with the posture of flexed hand fingers than extended hand fingers (Fig. 1B).

## Experiment 3

We analyzed the effects of previously assumed mouth postures (open and closed mouth) on the finger shaping during a successive hand grasp of objects.

### Methods

#### Participants

A new sample of ten right-handed [27] naïve volunteers (8 females and 2 males, age 23–28 yrs.) participated in the experiment.

#### Apparatus, Stimuli, and Procedure

The participants sat in front of a table on which they placed their right hand with the thumb and index finger in pinch position (Starting Position, SP). One of two wooden parallelepipeds (small target: 3×3×1 cm; large target: 5×5×1 cm) was placed on the table plane at a distance of 22 cm from SP. The participants were required to reach and grasp the presented parallelepiped with their right thumb and index finger, as shown in Figure 1C; they were required to move with a natural velocity as during spontaneous movements. The action was executed in three experimental conditions during which the mouth posture randomly varied. The postures were the following: open mouth, relaxed mouth and closed mouth. Before each trial, the participants, whose eyes were closed, were required to take one of the three mouth postures. When they were confident to have assumed the correct posture, they opened their eyes and started the reach to grasp action maintaining that mouth posture during the entire action. Apparatus, stimuli, and movements are shown in Figure 1C. The remaining procedure was the same as in experiment 1.

#### Data recording

The system of movement recording and analysis was the same as in experiment 1.We used three markers attached to the tip of the index finger, the thumb, and to the wrist of the participants' right hand. Another two markers were attached, one to the upper, and one to the lower lip of the participant. The markers placed on the thumb and the index finger were used to study the kinematics of the grasp. Grasp time course started with the hand in pinch position, and was constituted by a finger opening phase until a maximum (maximal finger aperture) followed by a phase of finger closing on the object [28]. We analyzed peak velocity of finger opening, and maximal finger aperture. The kinematics of the marker placed on the wrist was used to study the hand reach. We analyzed arm reach peak velocity. The markers placed on the upper and lower lips of the participants were used to measure the mouth aperture averaged across the hand reach motor act, i.e. from the reach beginning to the reach end. Data for two participants were discarded due to technical problems.

#### Data analysis

Separate ANOVAs were carried out on the mean values of the hand reaching-grasping parameters and mouth aperture. The within-subjects factors were target size (large versus small) and mouth posture (open mouth versus relaxed mouth versus closed mouth).

### Results and Discussion

**Figure 3:**
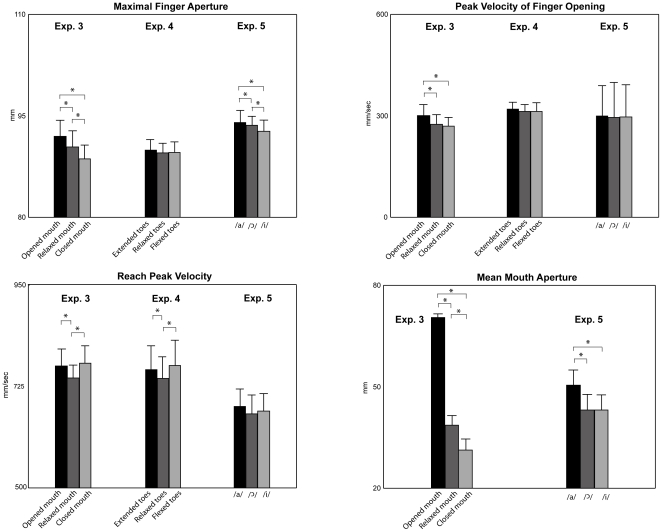
Mean values of parameters of hand grasp, hand reach and mean mouth aperture in the three experimental conditions of mouth posture in experiments 3, foot posture in experiment 4, and vocal pronunciation in experiment 5. Bars are SE. Asterisks indicate significance in the ANOVAs.

Peak velocity of finger opening and maximal finger aperture were affected by mouth posture (F(1,9) = 3.4, p = 0.05, η^2^_p_ = 0.27, F(1, 9) = 7.2, p<0.0001, η^2^_p_ = 0.44, Fig. 3). These parameters were greater in the condition of open mouth as compared to the conditions of closed and relaxed mouth (post-hoc comparison). Maximal finger aperture was also significantly greater in the condition of relaxed mouth as compared to closed mouth condition (post-hoc comparison). Reach peak velocity was significantly greater in the two conditions of open and closed mouth as compared to the condition of relaxed mouth (F(1, 9) = 3.9 p<0.05, η^2^_p_ = 0.30; Fig. 3, post-hoc comparison). No significant difference was found between the conditions of open and closed mouth (post-hoc comparison). Mean mouth aperture significantly differed in the three conditions of mouth posture: it significantly increased moving from closed mouth to open mouth conditions (F(1, 7) = 78.1, p<0.0001, η^2^_p_ = 0.91, Fig. 3, post-hoc comparison). The other results are reported in Table S3 (Results of ANOVAs on kinematic parameters of reaching and grasping executed with the hand when the mouth is opened, relaxed and closed) and data concerning experiments 1–3 are discussed in Text S1 (Effects of target size on head reach and hand reach).

The effects of the mouth postures on finger shaping during the hand grasp were more consistent as those of the hand postures on lip shaping during the mouth grasp (experiments 1–2). Indeed, an effect was also observed on peak velocity of finger opening and the effect of relaxed hand posture was significant different from the effects of opened and closed mouth. To explain these results we assume that the hand grasp was likely more automatic and its execution was less controlled than the mouth grasp. Consequently, the effects of the mouth postures on the grasp planning were not attenuated by the control of movement execution. This, on the contrary, occurred for the more controlled execution of grasp with the mouth because approaching the target with the head is less habitual than approaching the target with the hand. Note that in experiments 1 and 2 the peripheral visual control of the hand posture was allowed during grasp execution, whereas in experiment 3 the visual control of mouth posture was not possible. Nevertheless, the effects of mouth postures were more consistent than those of hand postures in experiments 1 and 2. This suggests that the hand posture was scarcely controlled with peripheral vision during execution of mouth grasp.

## Experiment 4

We analyzed the effects of foot postures on finger shaping during grasps of objects with the hand. We chose as foot postures toe extension/flexion, which may be associated to interactions of the distal part of foot with objects and were comparable with the postures assumed by the other distal effectors in experiments 2 and 3.

### Methods

#### Participants

A new sample of nine right-handed [27], naïve volunteers whose right feet were prehensile (5 females and 4 males, age 22–27 yrs.) participated in the experiment.

#### Apparatus, Stimuli, and Procedure

Apparatus and stimuli were the same as in experiment 3. The reaching-grasping action was executed in three experimental conditions during which one of the following postures of the right foot was assumed before and maintained during hand action execution: extended, relaxed or flexed toes. The foot heel rested on the floor. Consequently, in flexed toe posture the foot was slightly dorsi-flexed. The remaining procedure was the same as in experiment 3.

#### Data Recording and Analysis

Hand movement recording and analysis were the same as in experiment 3. In the ANOVAs the within-subjects factors were target size (large versus small) and foot posture (extended versus relaxed versus flexed toes). During the experimental session the posture of the right foot was recorded by means of a video camera because, using the SMART system, hand movements and foot postures could be simultaneously recorded with less spatial resolution as compared to the other experiments of the present study. For each trial we verified whether the foot posture was correctly taken before and maintained during the successive reaching-grasping action.

### Results and Discussion

Foot posture did not affect the grasp kinematics whereas it affected reach peak velocity. Reach peak velocity increased in the two conditions of extended and flexed toes as compared to relaxed foot (F(1, 8) = 3.8, p<0.05, η^2^_p_ = 0.32, Fig. 3, post-hoc comparison ). No significant difference was found between the conditions of extended and flexed toes (post-hoc comparison). The analysis of the recording by means of the video camera showed that the foot postures were correctly taken in all trials. The other results are reported in Table S4 (Results of the ANOVAs on kinematic parameters of reaching and grasping executed with the hand while the toes are extended, relaxed and flexed).

The foot postures did not affect the finger shaping during the hand grasps of objects. In contrast, reach peak velocity increased during the postures of both extended and flexed toes in the comparison with the posture of relaxed toes. The same results were found in experiment 3. However, the type of posture did not modulate the reach parameters.

## Experiment 5

We examined whether vocalization affects the simultaneous grasp of objects: we required participants to vocalize and then to grasp objects of different size while continuing to vocalize. We considered that vocalizations require particular postures of the internal mouth [29]–[30]. Open vowels, such as /a/, are related to large internal mouth apertures, whereas closed vowels, such as /i/, are related to small internal mouth apertures. If mouth postures affect the control of grasp (experiment 3), it is possible that even specific internal mouth postures required for the pronunciation of vowels affect the control of grasp.

### Methods

#### Participants

A new sample of ten right-handed [27], naïve volunteers (5 females and 5 males, age 25–30 yrs.) participated in the experiment.

#### Apparatus, Stimuli, and Procedure

Apparatus and stimuli were the same as in experiment 3. The reaching-grasping was executed after the participants started to pronounce one of the three following vowels: /a/, 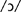 and /i/. We chose 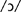 as control vocalization because its internal mouth aperture during vowel production is intermediate between the internal mouth apertures during /a/ and /i/ vocalizations. During the reaching-grasping action the participants continued to vocalize. The remaining procedure was the same as in experiment 3.

#### Data Recording and Analysis

Recording of hand movements and mouth posture was as in experiment 3. Moreover, the participants wore a light-weight dynamic headset microphone (Shure, model WH20). The frequency response of the microphone ranged from 50 to 15,000 Hz. The microphone was connected to a PC by a sound card (16 PCI Sound Blaster; CREATIVE Technology Ltd, Singapore). We acquired voice data during vowel pronunciation using the Avisoft SASLab professional software (Avisoft Bioacoustics, Germany), whereas we calculated the participants' voice parameters using the PRAAT software (www.praat.org). We calculated mean values of formant (F) 1 and 2 during reach execution. Note that F1 and F2 univocally define each vowel from an acoustical point of view [30]. Data for one participant were discarded because of noisy acquisition. Kinematic and vocal parameters were submitted to ANOVAs, the within-subjects factors of which were target size (large versus small) and vowel (/a/ versus 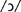 versus /i/).

### Results and Discussion

Maximal finger aperture (F(1, 9) = 6.5, p<0.01, η^2^_p_ = 0.40) and mean mouth aperture (F(1, 9) = 20.0, p<0.001, η^2^_p_ = 0.69) were affected by vowel pronunciation. Maximal finger aperture was significantly greater in the condition of /a/ pronunciation as compared to the conditions of 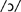 and /i/ pronunciation, and greater in the condition of 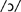 as compared to /i/ pronunciation (Fig. 3, post-hoc comparison). Mean mouth aperture was greater in the condition of /a/ pronunciation as compared to 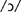 and /i/ pronunciation (Fig. 3, post-hoc comparison). No significant difference was found between 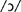 and /i/ pronunciation (post-hoc comparison).

**Figure 4:**
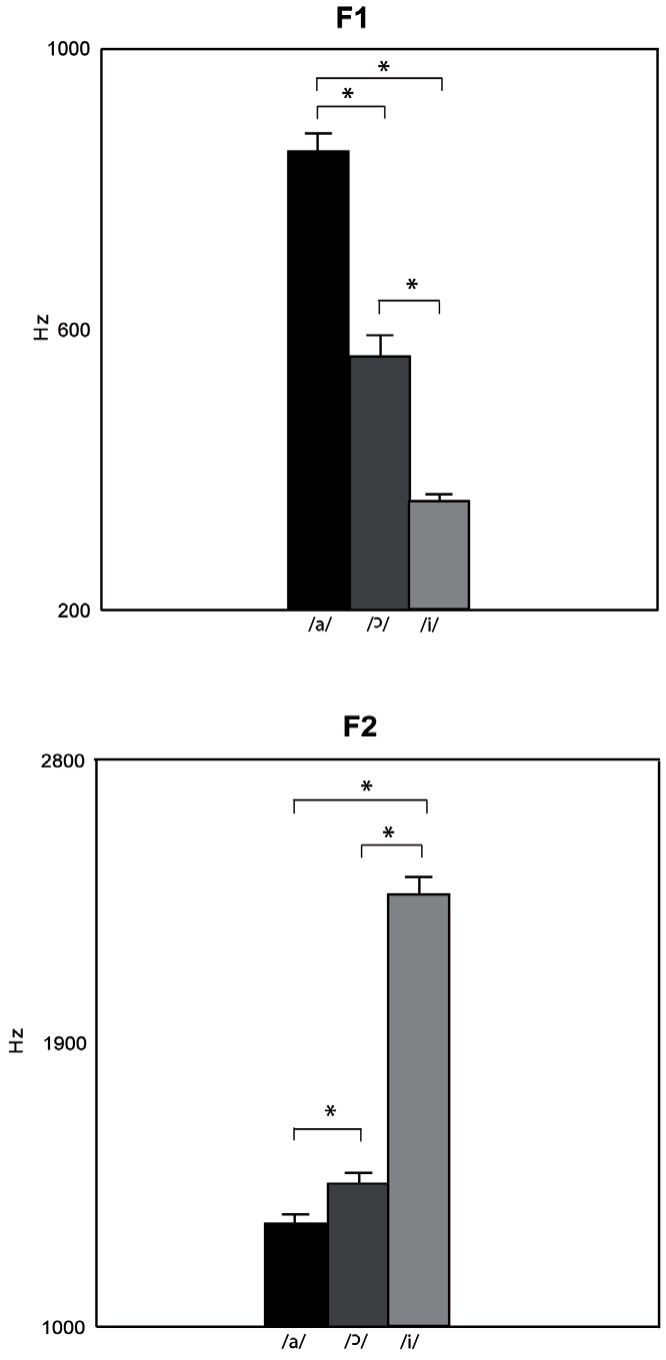
Mean values of vocal parameters of the three vocalizations averaged across reach execution in experiment 5. Bars are SE. Asterisks indicate significance in the ANOVAs.

F1 (F(1, 8) = 235.5,p<0.0001, η^2^_p_ = 0.96) and F2 (F(1, 8) = 283.6, p<0.0001, η^2^_p_ = 0.97) significantly differed in the three conditions of vowel pronunciation. F1 significantly increased, whereas F2 significantly decreased moving from closed to open vowels (/i/ versus 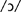 versus /i/, Fig. 4, post-hoc comparison). The other results are reported in Table S5 (Results of the ANOVAs on kinematic parameters of manual reaching and grasping and voice parameters while pronouncing /a/, 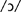, and /i/ during movement execution).

The pronunciation of vowels affected the finger shaping during the simultaneous grasp of targets. Moving from closed to open vowels (/i/ versus 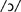 versus /a/) there was a significant gradual increase in finger shaping. This result cannot be attributed to external mouth aperture only (lip aperture) because this parameter increased in the condition of /a/ pronunciation as compared to 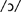 and /i/ pronunciation. In contrast, the voice spectra parameters significantly varied among the three vowel pronunciations. In particular, F1 significantly increased, moving from close to open vowels (/i/ versus 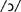 versus /a/, Fig. 4). F1 is mainly related to aperture of the internal mouth [30]. However, because of the difficulty of a precise recording, we cannot relate the variation in finger shaping to postures of specific articulatory organs of mouth involved in phoneme production (namely, the lips, the velum, the larynx, and the blade, body, and root of the tongue) [29]. These organs are responsible for variation in mouth configurations and, consequently, for variation in vocal parameters.

## General Discussion

In experiments 1 and 2, hand postures affected the kinematics of grasps with the mouth; conversely, in experiment 3 mouth postures affected the kinematics of grasps with the hand. In contrast, in experiment 4 foot postures had no effect on grasps with the hand. These results confirm the strict relation between hand and mouth [26]. Previously, we proposed that commands of grasp with the hand are also sent to the mouth and vice versa commands of grasp with the mouth are also sent to the hand. This proposal is in agreement with the discovery of neurons in monkey F5 premotor cortex which discharge when the animal grasps an object with the hand or the mouth [3]. The results of the present study show that functional relations between distal effectors occur at level of their postures in addition to their movements; specifically, the posture of one effector (the mouth or the hand) can be a template for the configuration that will be reached by the other grasping effector (the hand or the mouth) during shaping. In particular, the configurations of power grip and precision grip (experiment 1) and the postures of finger extension and flexion (experiment 2) were transferred to the grasping mouth as commands of enlarging and shortening the lip shaping. Conversely, the postures of open and closed mouth were transferred to the grasping hand as commands of enlarging and shortening the finger shaping. Since these postures were assumed before the movement, the possibility of a temporal coupling between the openings of the two effectors is excluded. In fact, these postures probably affected the visuo-motor transformation performed by the grasping effector. This process took place after the postures were assumed. Concerning the hand postures, the data from experiment 2 suggest that the simple finger extension/flexion is sufficient to affect the mouth grasp. This is plausible if we consider that the grasp with the mouth is mainly constituted by a lowering/lifting of the jaw; these movements are associable with finger extension/flexion.

In experiment 4, the extension/flexion of the toes did not affect the grasp with the hand. However, evidence [31] does support the hypothesis that the control of hand movements can be associated to the control of foot movements, suggesting a synchrony of coupled hand-foot movements. To explain this apparent contradictory result, firstly we should consider that, in the present study, we analyzed the effects of postures of an effector on the action planning with another effector rather than the synchrony of coupled movements of different distal effectors. Secondly, in modern humans the foot has lost the capacity of activating different types of interactions with objects of different size and shape. For this reason, in a task in which the type of interaction with objects is implicitly tested, hand and foot do not interact with each other, as hand and mouth do because different types of interaction with objects can be activated by both the hand and mouth only. Neuroimaging data [32] support this possibility. Indeed, they show that premotor area where foot actions are planned is separated from premotor area involved in planning of hand actions. In contrast, hand and mouth areas are adjacent and partially overlap.

The transfer of postures of an effector to movements of another effector was restricted to distal movements (hand and mouth). In fact, the reach (proximal/axial) component was not modulated by the different postures taken by the other distal effector as the grasp was. In fact, in experiment 3, the arm reach was faster when the mouth musculature was contracted (opened and closed mouth) in comparisons with the relaxed mouth posture. Moreover, in experiment 4, the contraction of foot muscles (extended and flexed foot fingers) affected the arm reach as in experiment 3 without affecting the hand grasp. The proximal/axial muscle activations, however, were not modulated the type of posture taken by the distal effector. Consequently, these results may be explained as due to unspecific activations of proximal/axial muscles and distal muscles.

Previously, Gentilucci and colleagues [26] found that the control of grasp movements affects the production of phonemic units. On the basis of these results the authors proposed that during evolution double commands of grasp with hand and mouth were used to transfer a communication system based on arm gestures to a mouth articulation gesture system, which were later co-opted for speech [17]–[19]. The system of double commands could also be the basis on which the reciprocal interactions between speech and gestures were constructed [20]–[22]. However, to be validated, this hypothesis required verification of whether the production of phonemic units influences the control of grasp movements. This was verified in experiment 5. The results showed that vocalizations influenced grasp movements. Specifically, production of /i/, 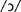 and /a/ induced a significant gradual increase in finger shaping. Correspondingly, the vocal parameters significantly varied during the three vocalizations showing a gradual increase (or decrease) moving from closed to open vowels (see Fig. 4).

Moreover, experiment 5 tried to solve an unclear aspect concerning language evolution: i.e. how abstract symbols (i.e. the words) could become associated with aspects of the real word. One theory proposed by Paget [33], called “schematopoeia”, holds that spoken words arose initially from parallels between sound and meaning. For example, in modern languages vowels are frequently open in words coding something large, but are closed in words coding something small (*gr/a/nd*e vs. *p/i/ccolo*; *gr/a/nd* vs. *pet/i/t*; note that “a” is differently pronounced in the words *large* and *small*). The results of the present study may partially support this theory: /a/ as compared to /i/ induced larger finger apertures corresponding to a motor coding of a larger object. Indeed, it is well known that maximal finger aperture increases with increasing in object size [34] [28].

Summing up, the results of the present study support the hypothesis of the existence of interactions between mouth and hand. Specifically, postures assumed by an effector congruently affect the shaping of the other grasping effector. The results go beyond a simple temporal coupling between movements of the two distal effectors, but they indicate that kinematic parameters related to the posture of an effector are transferred to the other one when planning an action. These effects are specific for hand and mouth, rather than foot. Vocalizations affect the control of grasp, as conversely the control of grasp affects production of phonemic units [26]. The processes inducing these effects may be at the basis of the construction of interactions between gestures and words [20]–[22] and the system producing these effects may have been used to transfer a communication system based on arm gesture to a communication system based on mouth gestures during language evolution [17]–[19]. Finally, vowels seem to be involved in coding physical features of objects, and, more in general, they seem to be related to aspects of the external word.

## Supporting Information

Table S1 (DOC)

Table S2 (DOC)

Table S3 (DOC)

Table S4 (DOC)

Table S5 (DOC)

Text S1 (DOC)
