# Supplementary material for: Do Postures of Distal Effectors Affect the Control of Actions of Other Distal Effectors? Evidence for a System of Interactions between Hand and Mouth
Source: PLoS One. 2011 May 23;6(5):e19793. doi: 10.1371/journal.pone.0019793 (PMC3100300; doi:10.1371/journal.pone.0019793)
Supplement: Table S1 — (DOC) [file pone.0019793.s001.doc]

|  | **Table S1. Results of the ANOVAs on kinematic parameters of reaching and grasping executed with the mouth while the hand is in a power grip posture, is relaxed, and is in a precision grip posture.** | | |
| --- | --- | --- | --- |
|  | **EXPERIMENT 1** | | |
|  | ***Object size***  ***Large versus small*** | ***Hand posture***  ***Power grip versus relaxed hand versus precision grip*** | ***Object size x hand posture*** |
| **Peak velocity of lip opening**  **(mm/sec)** | F(1,9)=11.2,  p<0.01, η^2^p=0.55;  72.5 versus 67.0 | F(1,9)=0.05,  n.s. | F(2,18)=1.1,  n.s.; |
| **Maximal lip aperture**  **(mm)** | F(1,9)=51.8,  p<0.0001, η^2^p=0.85;  61.4 versus 59.1 | F(1, 9)=6.2,  p<0.001, η^2^p=0.41;  Fig.2 | F(2,18)=0.3,  n.s. |
| **Head reach peak velocity**  **(mm/sec)** | F(1,9)=4.8,  p=0.056,  400.2 versus 410.0 | F(1, 9)=0.1,  n.s. | F(2,18)=0.1,  n.s. |
| **Mean Finger aperture**  **(mm)** | F(1,8)=0.7,  n.s. | F(1, 8)=78.4,  p<0.0001, η^2^p=0.90;  Fig.2 | F(2,16)=0.03,  n.s. |
