# Supplementary material for: Do Postures of Distal Effectors Affect the Control of Actions of Other Distal Effectors? Evidence for a System of Interactions between Hand and Mouth
Source: PLoS One. 2011 May 23;6(5):e19793. doi: 10.1371/journal.pone.0019793 (PMC3100300; doi:10.1371/journal.pone.0019793)
Supplement: Table S2 — (DOC) [file pone.0019793.s002.doc]

|  | **Table S2. Results of the ANOVAs on kinematic parameters of reaching and grasping executed with the mouth while the hand fingers are extended, relaxed and flexed.** | | |
| --- | --- | --- | --- |
|  | **EXPERIMENT 2** | | |
|  | ***Object size***  ***Large versus small*** | ***Hand posture***  ***Extended versus relaxed versus flexed fingers*** | ***Object size x hand posture*** |
| **Peak velocity of lip opening**  **(mm/sec)** | F(1,9)=1.1,  n.s. | F(1,9)=0.1,  n.s. | F(2,18)=0.5,  n.s.; |
| **Maximal lip aperture**  **(mm)** | F(1,9)=40.0,  p<0.0001, η^2^p=0.81;  66.9 versus 62.7 | F(1, 9)=3.9,  p<0.05, η^2^p= 0.30;  Fig.2 | F(2,18)=3.3,  P=0.06; |
| **Head reach peak velocity**  **(mm/sec)** | F(1,9)=0.1,  n.s. | F(1, 9)=2.0,  n.s. | F(2,18)=0.4,  n.s. |
| **Mean Finger aperture**  **(mm)** | F(1,8)=0.4,  n.s. | F(1, 8)=12.6,  p<0.0001, η^2^p=0.58;  Fig.2 | F(2,18)=0.3,  n.s. |
