# Supplementary material for: Do Postures of Distal Effectors Affect the Control of Actions of Other Distal Effectors? Evidence for a System of Interactions between Hand and Mouth
Source: PLoS One. 2011 May 23;6(5):e19793. doi: 10.1371/journal.pone.0019793 (PMC3100300; doi:10.1371/journal.pone.0019793)
Supplement: Table S4 — (DOC) [file pone.0019793.s004.doc]

|  | **Table S4. Results of the ANOVAs on kinematic parameters of reaching and grasping executed with the hand while the toes are extended, relaxed and flexed.** | | |
| --- | --- | --- | --- |
|  | **EXPERIMENT 4** | | |
|  | ***Object size***  ***Large versus small*** | ***Posture of toes***  ***Extended versus relaxed versus flexed*** | ***Object size x toe posture*** |
| **Peak velocity of finger opening**  **(mm/sec)** | F(1,8)=15.9  p<0.005, η^2^p=0.66;  337.4 versus 277.6 | F(1,8)=0.9,  n.s. | F(2,16)=0.1,  n.s.; |
| **Maximal finger aperture**  **(mm)** | F(1,8)=251.2,  p<0.0001, η^2^p=0.96;  97.1 versus 81.6 | F(1, 8)=0.5,  n.s. | F(2,16)=1.4,  n.s. |
| **Arm reach peak velocity**  **(mm/sec)** | F(1,8)=1.9,  n.s. | F(1, 8)=3.8,  p<0.05, η^2^p=0.32;  Fig.3 | F(2,16)=2.9,  n.s. |
