# Supplementary material for: Do Postures of Distal Effectors Affect the Control of Actions of Other Distal Effectors? Evidence for a System of Interactions between Hand and Mouth
Source: PLoS One. 2011 May 23;6(5):e19793. doi: 10.1371/journal.pone.0019793 (PMC3100300; doi:10.1371/journal.pone.0019793)
Supplement: Table S5 — (DOC) [file pone.0019793.s005.doc]

|  | **Table S5. Results of the ANOVAs on kinematic parameters of manual reaching and grasping and voice parameters while pronouncing /a/, /*ɔ*/, and /i/ during movement execution.** | | |
| --- | --- | --- | --- |
|  | **EXPERIMENT 5** | | |
|  | ***Object size***  ***Large versus small*** | ***Vowel***  ***/a/ versus /*ɔ*/ versus /i/*** | ***Object size x Vowel*** |
| **Peak velocity of finger opening**  **(mm/sec)** | F(1,9)=18.1,  p<0.001, η^2^p=0.66;  314.3 versus 280.9 | F(1,9)=0.1,  n.s. | F(2,18)=0.3.,  n.s.; |
| **Maximal finger aperture**  **(mm)** | F(1,9)=127.1,  p<0.0001, η^2^p=0.93;  101.0 versus 85.9 | F(1, 9)=6.5,  p<0.01, η^2^p=0.40;  Fig.3 | F(2,18)=0.1,  n.s. |
| **Arm reach peak velocity**  **(mm/sec)** | F(1,9)=0.1,  n.s. | F(1, 9)=1.4,  n.s.  Fig.3 | F(2,18)=0.5,  n.s. |
| **Mean Mouth aperture**  **(mm)** | F(1,9)=0.5,  n.s. | F(1, 9)=20.0,  p<0.001, η^2^p=0.69;  Fig.3 | F(2,18)=0.4,  n.s. |
| **Formant 1**  **Hz** | F(1,8)=02.,  n.s. | F(1, 8)=235.5,  p<0.0001, η^2^p=0.96;  Fig.4 | F(2,16)=0.4,  n.s. |
| **Formant 2**  **Hz** | F(1,8)=0.7,  n.s. | F(1, 8)=283.6,  p<0.0001, η^2^p=0.97;  Fig.4 | F(2,16)=0.6,  n.s. |
