# Supplementary material for: Do Postures of Distal Effectors Affect the Control of Actions of Other Distal Effectors? Evidence for a System of Interactions between Hand and Mouth
Source: PLoS One. 2011 May 23;6(5):e19793. doi: 10.1371/journal.pone.0019793 (PMC3100300; doi:10.1371/journal.pone.0019793)
Supplement: Text S1 — (DOC) [file pone.0019793.s006.doc]

In experiments 1 and 2, the head reach slowed down when interacting with the large object (see Tables S1-S2). This result seems to be in contrast with the results concerning the reaches to grasp executed with the hand found in experiment 3 during which, on the contrary, the hand reach slows down when interacting with small objects (see Table S3). This apparent contradiction can be explained by considering that the index of difficulty [Fitts PM (1954) The information capacity of the human motor system in controlling the amplitude of movement. J Exp Psychol Hum Percept Perform 47: 381-391] probably increased, (inducing a movement slowing down) when grasping larger object because of the limited possibility of mouth aperture with respect to increases in object size. This was unlike to occur when the grasp was executed with the hand.
