# Supplementary material for: Do Postures of Distal Effectors Affect the Control of Actions of Other Distal Effectors? Evidence for a System of Interactions between Hand and Mouth
Source: PLoS One. 2011 May 23;6(5):e19793. doi: 10.1371/journal.pone.0019793 (PMC3100300; doi:10.1371/journal.pone.0019793)
Supplement: Supplementary file 6 [file pone.0019793.s003.doc]

Table S3.  Results of ANOVAs on kinematic parameters of reaching and grasping executed with the hand when the mouth is opened, relaxed and closed. 			EXPERIMENT 3			Object size
Large versus  small	Mouth posture
Opened versus relaxed versus closed
	Object size x Mouth posture		Peak velocity of finger opening
(mm/sec)	F(1,9)=11.6
p<0.01, η2p=0.56;
306.4 versus 267.5	F(1,9)=3.4, 
p=0.05, η2p=0.27;
Fig.3	F(2,18)=0.5, 
n.s.; 

		Maximal finger Aperture
(mm)	F(1,9)=156.0, 
p<0.001, η2p=0.94;
98.0 versus 88.7	F(1, 9)=7.2, 
p<0.0001, η2p=0.44;
Fig.3	F(2,18)=2.2, 
n.s.		Arm reach peak velocity
(mm/sec)	(1,9)=4.5, 
p=0.06,
772.5 versus 755.2	F(1, 9)=3.9,
p<0.05, η2p=0.30;
Fig.3	F(2,18)=1.0, 
n.s.		Mean Mouth aperture
(mm)	F(1,7)=3.0, 
n.s.	F(1, 7)=78.1, 
p<0.0001, η2p=0.91;
Fig.3	F(2,14)=2.9, 
n.s.
